# Plausibly deniable - Domestic cannabis cultivation and the private rented sector in the UK

**DOI:** 10.1007/s12117-025-09571-7

**Published:** 2025-07-17

**Authors:** Xavier L’Hoiry, Julie Rugg, Loren E. Parton, Georgios A. Antonopoulos

**Affiliations:** 1 School of Sociological Studies, Politics and International Relations, University of Sheffield, Sheffield, UK; 2 School for Business and Society, University of York, York, UK; 3 Centre for Crime & Policing, Department of Social Sciences, Northumbria University, Newcastle upon Tyne, UK

**Keywords:** Domestic cannabis cultivation, Private rented sector, Landlords, Organised crime

## Abstract

Domestic cannabis cultivation has grown exponentially for the past several decades and is said to outstrip importation from abroad in many jurisdictions, the UK included. While research has been attentive to this shift, scant attention has been paid to the space in which much domestic cannabis cultivation takes place: privately rented residential property. This article explores how and why the private rented sector in the UK facilitates and incubates cannabis cultivation and makes this an attractive space for criminal actors to partake in this illegal activity. Drawing on 43 qualitative interviews with police and local authority practitioners in the UK with experience of intervening in domestic cannabis cultivation, the article details the many affordances of the private rented sector for those involved in cannabis cultivation. Key among these affordances are the layers of plausible deniability available to several of the actors involved– landlords, letting agents, tenants/growers– which serve to frustrate and obfuscate police investigations. The article also examines the instrumental role of residential property itself as a critical but easily disposable commodity which facilitates cannabis cultivation.

## Introduction

Global seizures of cannabis have increased steadily across the past 20 years and the trafficking of cannabis across international borders remains a key facet of this illicit marketplace (Mejdini 2022; UNODC 2025). In the past three decades however, a considerable shift towards domestic cultivation of cannabis has been documented by academic research, with some arguing that domestic cultivation now accounts for most of the cannabis consumed in many countries (see Decorte 2010; Barratt et al. 2012). The underlying logic of the move towards domestic cultivation is that cannabis, unlike many other illegal drugs, can be grown anywhere in the world under the right conditions. The affordability and wide accessibility of agronomic technologies (Ancrum and Treadwell 2016; Decorte 2010), the availability online of guidance on how to grow cannabis (Potter 2008), and the ‘minimal processing’ (Barratt et al. 2012) required before cannabis can be consumed have led to what Decorte (2010: 271) has described as an ‘irreversible international trend’ towards domestic cultivation. As Kirby and Peal (2015: 280) have explained, domestic cultivation of cannabis has become:

‘operationally and financially viable as it reduce(s) the number of people that [are] needed to be involved, thereby reducing the risk of detection as well as lowering the handling and transportation costs’.

This shift, described as an ‘import substitution’ (Jansen 2002), has ushered in what Potter and Klein (2020: 201) have called the ‘democratisation of the cannabis market’, with a vast range of actors involved in cultivating cannabis, from organised crime groups to enthusiastic amateurs (Potter 2008). While extant literature has documented several facets of domestic cannabis cultivation in the UK and elsewhere, exploring actors’ motivations, logistical operations and detection avoidance strategies, there has been scant academic attention to a key space in which such activity takes place: privately rented residential accommodation. Moreover, little consideration has been given to the role of property itself in supporting cannabis cultivation. This article draws on interviews with law enforcement and other practitioners with occupational experience of intervening in domestic cannabis cultivation to explore the ways in which the private rented sector (PRS) offers a series of affordances to actors involved in cannabis cultivation, making this a particularly attractive space in which to carry out this illegal activity. Key to our argument is the particular affordance of plausible deniability, which pervades the PRS and allows criminal actors to deny and obfuscate their involvement in cannabis cultivation. Private rental constitutes a relationship between landlords, tenants and often letting agents, who all might be involved at various levels in cultivation but where the rental relationship means that blame can easily be transferred. Alongside this, the article also considers how the spatiality of the PRS presents ample opportunities for criminality, including the instrumental role of property itself, as well as the neighbourhoods in which much cannabis cultivation takes place. These are often socio-economically marginalised, experiencing various forms of disenfranchisement, high levels of residential mobility, and a tendency towards minimal engagement with police. The approach taken in this article makes original contributions to existing literatures in this field. By centring the role of property in domestic cannabis cultivation, the article adds a new perspective to discussions which have generally tended to focus on the personal motivations of actors in this context rather than the instrumental affordances of certain premises. In situating cannabis cultivation as an example of criminality taking place in the PRS and focusing on the PRS as a space which supports this type of illegal activity, the article also advances criminological literatures more broadly. Literatures have largely ignored the role of the PRS in incubating certain forms of crime, despite non-criminological research increasingly drawing attention to this (see Spencer et al. 2020; Rugg, in press). While some previous research has been attentive to property and housing as they relate to some types of drug-related criminality (such as cuckooing (see Spicer 2024), the specificity of housing tenure tends to be overlooked. Privately rented residential premises, and the PRS more broadly, therefore, remain largely beyond the gaze of criminological research and this article directly addresses this omission.

The article is set out in five parts following this introduction. First, the article details key elements of the PRS in the UK, outlining the types of landlords involved in this context and the presence of other actors such as letting agents. Second, we situate our discussions within existing literature on domestic cannabis cultivation, particularly work which has explored this phenomenon in the UK. Third, the methods which underpinned this study are outlined, including descriptions of the sampling strategy, recruitment, fieldwork and data analysis processes. The fourth section presents primary data which draws attention to the role of property and the PRS in the context of cannabis cultivation, reflecting on the challenges for intervention in this context and, by extension, the benefits enjoyed by criminal actors operating in this space. The article concludes by arguing that the PRS represents an ideal environment in which to participate in this illegal activity. Unlike cannabis farming in social renting, where only the tenant is likely to be culpable, the private rented sector constitutes space in which tenants, landlords and letting agents– separately or working in operation– can all be involved in cannabis farming and use difficulties around evidencing culpability to thwart police investigation.

## The private rented sector, landlords and letting agents in England^1^

After a protracted period of decline, the PRS in England began to grow from 2000, exceeding the size of the social rented sector in 2011/12; the sector currently accommodates 18.8 per cent of all households in England (EHS 2023). This part of the housing market covers a wide range of niche markets catering for specific demand groups: student housing is perhaps the most familiar submarket (Rugg and Rhodes 2018). Around a third of the PRS can be defined as being ‘lower end’, that is with a tenant wholly or partly reliant on benefit and/or paying a below-average rent and/or with an income in the lowest deciles (Rugg and Wallace 2021).

Landlordism in England is frequently characterised as a ‘cottage industry’, given the large number of landlords with very small residential portfolios. Over 90 per cent of landlords operate as private individuals, and 45 per cent have just one property (MHCLG 2024). This means that practices in the sector can be associated with amateurish behaviour, with landlords being unaware of their legal responsibilities (DLUHC 2022). Indeed, some individuals may not necessarily characterise themselves as landlords, since their letting might comprise an informal agreement with a friend or relative. That having been said, in 2021 over 50 per cent of landlords had been letting for eleven years or more. Landlords let property in various circumstances and their activity is often dynamic: they move into, within and out of the PRS, with property purchase often reflecting life course experience and external factors including regulatory and financial change.

In qualitative study of the sector, Rugg and Wallace (2021) characterised four landlord types according to intent and modes of operation. First, ‘accidental’ landlords acquire property without the intention of letting it out: it may be a ‘spare’ property when relationships form between two homeowners, or an inheritance let as an interim measure. This type of landlord tends not to stay in the rental market for a protracted period. Second, ‘investment’ landlords work full or part-time and purchase property as the most effective use of disposable income, often with the expectation that the property will provide a pension in later life either through rental income or outright sale. Their portfolio might comprise 1–10 properties, and decisions generally reflect the desire to maximise tax efficiency. Neither of these types of landlords will be giving full-time attention to their property (Rugg and Wallace 2021).

Third, full-time landlords are engaged in letting property as their only employment and principal source of income. Their business might be run as a family business, with property holdings extending to partners and adult children for tax purposes. These landlords can often be ‘hands on’ in managing their portfolio, attending personally to rent collection, property development, repair and maintenance. They often develop their portfolio in a geographically concentrated area, for ease of access. For full-time landlords, portfolio size can be substantial and run into dozens of properties which means the landlord might share the burden of management with family members. Fourth, business landlords have a property portfolio in addition to other commercial interests, which might not be related to property. This type of landlord might switch capital between businesses as required. Business landlords often employ managers specifically to handle their portfolios.

Letting agents offer a range of services that are generally classified as ‘letting’ and ‘management’. An agent might offer a simple ‘find a tenant’ service, in arranging a property to be advertised, vetting tenants and setting up the initial tenancy. The agent might or might not then continue to manage the property on behalf of the landlord, often offering a range of services in return for a proportion of the rent. This kind of arrangement can benefit landlords who do not live close to their residential property, since regular property checks are generally included as a service. In 2024 it was reported that 43 per cent of landlords used an agent to let property, and 18 per cent used an agent to manage property (MHCLG 2024). As with landlords, there is substantial variety in the business modes of letting agents. Some are large-scale, branded organisations; some are attached to estate agents. Many are smaller, local ‘shop front’ arrangements with one or two staff, operating as small businesses much as many full-time landlords do. Some letting agents operate entirely on-line. In some instances, individual letting agents might also own property, and some landlords also manage property on behalf of other landlords.

Substantial variation in the character of landlords, motivations for letting and modes of letting create multiple opportunities for criminal enterprise. The size and highly fractured nature of the market stymies effective regulation. In England, around 1,600 social landlords let approximately four million tenancies, overseen by a social housing regulator. This compares with an estimated 2.82 million private landlords in England, letting around 4.6 million properties, with no similar overarching regulatory framework. In 2017, ONS data indicated that there were 15,705 PAYE (Pay As You Earn)-based enterprises managing real estate on a fee or contract basis.^2^

Private letting takes place within a complicated web of tenancy and property law, which defines a wide and perplexing array of letting arrangements (see, for example, Arden et al. 2012: 18ff). There is no legal obligation for a landlord to provide a tenancy agreement. A series of Housing Acts have defined property conditions and introduced licensing regimes for certain types of property. Enforcement in this regard is the purview of local authority private sector housing teams, generally located within environmental health departments (Stewart and Moffatt 2022). In 2021 the Chartered Institute of Environmental Health reported that a quarter of its members worked in housing, equating to just 856 full-time staff members. The Protection from Eviction Act 1977 defines harassment and illegal eviction as a criminal offence, although there is limited appetite for prosecution for contravention (Spencer et al. 2020). Letting agents are largely regulated by consumer protection law, but– as with environmental health officers– the number of Trading Standards staff is wholly inadequate to the task of effective regulation (NAO 2021).

## Domestic cannabis cultivation and the import substitution

While both outdoor and indoor cultivation of cannabis has increased in the past decade, United Nations Office on Drugs and Crime (UNODC) data show that indoor cultivation has grown at a much faster pace, with an estimated 20% increase between 2012 and 2022, as compared to a 7% increase in outdoor cultivation. Academic literature has documented the growth of domestic cannabis cultivation as taking place across the world, with studies exploring this phenomenon in Spain, Canada, Belgium, the Netherlands, Finland, Denmark, Turkey and many more besides (see Decorte 2010; Wouters 2008; Arana and Sanchez 2011; Athey et al. 2013; Akgul and Sever 2014).

In the UK, the National Crime Agency (2025) has described local cannabis cultivation as operating on ‘an industrial scale’ while Dame Carol Black’s independent report on drugs has speculated that ‘for herbal cannabis, domestic cultivation exceeds importation’ (Black 2020: 11). Previous police data have supported this assertion, with a 150% growth in detected commercial cannabis farms in the UK recorded between 2008/09 and 2010/11 (Association of Chief Police Officers 2012). That is not to say that cannabis importation no longer takes place, and historical trafficking routes from Balkan and North African regions to Western Europe continue to play a key role of this illicit market (Mejdini 2022; UNODC 2025). Indeed, the National Crime Agency (2025) has claimed that the legalisation of cannabis in some countries has in fact led to a growth in cannabis importation to the UK in recent years, identifying importation from Thailand as an example of this. Nonetheless, the alleged import substitution of cannabis in the UK has been described as ‘one of the unsung success stories of the UK agro-industry’ (Potter and Klein 2020) and has elicited several criminological studies seeking to understand how and why domestic cannabis cultivation now appears to form a habitual part of the cultivation landscape.

Silverstone (2010) and Silverstone and Savage (2010) have focused on the presence of organised crime groups in cannabis cultivation in the UK, particularly Southeast Asian (Vietnamese) groups which they argue have altered the marketplace and disrupted the involvement of ‘indigenous British criminals and enthusiastic amateurs’ (2010: 10). Kirby and Penna (2010) have similarly found evidence of Vietnamese involvement in cannabis cultivation in the UK, often operating in commercial rented premises. Vietnamese groups have been said to exploit vulnerable individuals, often precarious migrants, who are coerced into operating as growers or farmers (Silverstone 2010). Evidencing victimhood and modern slavery in this context however remains a complex challenge for law enforcement and other agencies (Ramiz et al. 2020). Kirby and Peal (2015) meanwhile, have argued that police data have previously shown a shift in the composition of actors involved in domestic cultivation from foreign nationals to British nominals. Though instructive to some extent, it is worth noting Kirby and Peal’s (2015) dataset is now over a decade old and more recent trends in domestic cultivation may therefore be absent in their analysis. For instance, news reports in the UK have drawn attention to the involvement of Albanian nationals in cannabis cultivation in recent years (see Edrich 2025; McGivern 2025; Hymas 2024). Notably, for the focus of this article, Kirby and Peal (2015: 284) report that the police data they examined showed that ‘the vast majority of cannabis farms were discovered in residential premises… with more [plants] detected within private residences than social housing’. This is one of the very few analyses of domestic cultivation to situate this activity within premise type, but the data is unfortunately not further unpacked to determine whether private residences were owner-occupied or rented. Kirby and Peal (2015) also describe the challenges faced by law enforcement in this context, including a lack of public reporting to the police and the legal status of grow shops that sell hydroponic and lighting equipment legally. Though police detection techniques have improved, including the use of infrared technology to identify heat sources, Kirby and Peal (2015), as well as others (see Collison 1995; Potter and Klein 2020), have pointed to the adaptability and innovation of growers to avoid detection, as well as the rapid replacement of detected cannabis grows with new sites taking their place.

Ancrum and Treadwell (2016: 69) meanwhile have focused on the involvement of ‘independent entrepreneurial criminals’ for whom ‘the prevailing imperative and ethic is nakedly economic and instrumental’ (2016: 72). Drawing on ethnographic engagement with cannabis cultivators, they emphasize poverty and social exclusion in post-industrial, disadvantaged inner cities as key to understanding the development of cannabis cultivation in the UK. The accounts of participants in their study refer to cannabis being grown in ‘attics, garages, flats, apartments and lock ups’ (2016: 73) as well as rented properties in rural locations which were used exclusively for the purpose of cultivating cannabis. The authors identify the use of rented rural properties as a recurring theme:

‘(S)everal times we met individuals who were willing to disclose cultivating supplies in rural locations, seemingly weighing up the benefits of quiet isolated locations (which were perceived as less susceptible to robbery by other criminals and less out of the gaze of the forces of law and order) against the potential increased scrutiny that could be encountered in quiet, close knit rural locates.’ (2016: 76).

Elsewhere, Gary Potter’s work (2008and 2010) has drawn attention to the ‘banal reality of home-based, small-scale production’ (Potter and Klein 2020) and has sought to challenge law enforcement and media narratives centred on large-scale cannabis cultivation operated by organised crime groups. Potter’s work has emphasized the breadth of actors involved in domestic cultivation in the UK, including those growing for personal use and social supply, social/commercial growers who supply ‘their social networks at least in part to supplement their income’, commercial growers who ‘grow to make money… selling to any potential customer’ (Potter and Klein 2020: 205), and medical growers who are ‘motivated mainly by the perceived therapeutic values of cannabis’ (Hough et al. 2003; ix). While the boundaries of these categories can be fluid and Potter acknowledges the presence of organised crime actors in the cannabis market, a recurring theme of Potter’s work is the depiction of domestic cannabis cultivation as involving predominantly small-scale grows operated by everyday actors, often with legitimate professions.

While offering rich accounts of the domestic cannabis cultivation landscape in the UK, what is broadly missing from this body of research is a specific focus on, firstly, the PRS as a space in which cannabis cultivators enjoy a series of affordances which support their illegal activities; and, secondly, the instrumental role of residential property itself as a commodity which facilitates cultivation of cannabis.

## Methods

This article is part of a wider study titled ‘Criminality in the private rented sector and co-producing solutions’. It considers the experiences of various stakeholders involved within the PRS including landlords, tenants, police and local authority representatives. This article draws on data collected from qualitative interviews with police officers of varying ranks and roles, as well as local authority representatives, all of whom were able to draw on their experiences of intervening in cases of domestic cannabis cultivation taking place in the PRS. The study was conducted across a specific geographical footprint in England which encompasses four police forces, one Regional Organised Crime Unit and 15 local authorities. It is from these organisations that the participants were recruited. The region includes several large cities, towns and rural locations. Of the four police forces, one is among the largest in the UK based on workforce size, two forces are mid-sized, and one is small.

A combination of purposive and snowball sampling was employed to recruit participants from police forces and local authorities with experience of intervening with criminality in the PRS. The study sought to capture a range of police ranks (from Police Constable through to Detective Chief Inspector) and roles as well as roles within local authorities which involve intervention in the PRS, such as Environmental Health Officers, Housing Officers, Serious and Organised Crime Coordinators and other relevant practitioners. The research team drew on existing professional networks with police forces in the first phase of participant recruitment. In the second phase of recruitment, snowball sampling was used to ask interview participants to connect the team with additional potential participants. In total, this article draws on 43 practitioner interviews of which 36 were conducted with police officers/staff and seven with local authority practitioners. Of the police interviewees, 27 were officers/staff working with local forces, seven were Regional Organised Crime Unit officers/staff and two interviewees held a national portfolio which included a focus on cannabis cultivation. Interviews were conducted between July 2023 and March 2024^3^.

Semi-structured, one-to-one interviews were conducted, and participants were asked to draw on their occupational experiences to discuss criminality taking place in the PRS. Participants were not specifically prompted to discuss cannabis cultivation but identified this themselves as a key challenge in the PRS. Once raised by participants, interview questions sought to unpack how cannabis cultivation takes place (including discussing the role of property itself) and the extent to which the conditions of the PRS facilitate or disrupts this type of criminal activity. All but two interviews were audio recorded and transcripts were produced. For the two non-recorded interviews, detailed notes were produced. Informed consent was obtained from all participants prior to commencing the interviews, which were conducted online or in-person depending in participants’ preference. As per the ethical protocol of the study, all transcripts and notes were anonymised with any identifiable information redacted. Participants were notified of this prior to the interview commencing and were also provided with information outlining how data may be used to support academic and other outputs.

Interview data were subjected to thematic analysis to identify common themes within the experiences recounted by the participants. The analysis followed structured steps drawing on Clarke and Braun’s (2013) phases of thematic analysis. First, familiarisation with the data was undertaken by listening to audio files once and reading transcripts several times. Second, several themes were identified from the data and, relevant to this paper, the theme of cannabis cultivation and the obfuscation of cannabis production was highlighted as a recurring theme. Transcripts were then re-analysed several times to review the identified themes and split these where necessary. Here, sub-themes were identified such as the involvement of landlords and letting agents in criminal activities and the policing response to such criminality. Participants’ data were then extracted to provide compelling accounts of cannabis cultivation and the affordances of the PRS to support such criminal activity.

Like all research, this study has some limitations. The geographical footprint of the study, while large and diverse, remains only a portion of the UK. This may have implications for generalisation although existing research and media coverage of cannabis cultivation suggests the accounts of practitioners below are reflected elsewhere in the country. As a qualitative study, the data below are of course the subjective reflections of practitioners and, like all qualitative studies, this should be born in mind. However, these limitations notwithstanding, the dataset in this study and the accounts detailed below are also a key strength of this paper. Little academic research has engaged directly with practitioners in and outside of the police to consider domestic cannabis cultivation. Even fewer studies have specifically considered this in the context of the PRS, marking the data below as an entirely original contribution to discussions of domestic cannabis cultivation and practitioners’ experiences of intervening in this context.

## Findings

This section draws on interview data with law enforcement and local authority practitioners whose accounts, reflecting on their experiences of intervening in domestic cannabis cultivation, support our argument that the PRS offers offenders a series of affordances which make this space an attractive environment in which to partake in cannabis cultivation. Of primary importance is the fluid relationship between tenants, landlords and letting agents, where the very nature of the tenure engenders responses including naivety, passive complicity and active criminality. It is worth noting that the affordances discussed below overlap extensively and the opacity of the PRS and the actors involved in criminal activity in this space creates significant challenges for law enforcement and other actors seeking to intervene.

### Landlord absenteeism and naivety

The nature of the PRS and the composition of landlords in this space often results in low levels of professionalism, reflected in absenteeism and naivety. As outlined above, many landlords may be characterised as ‘accidental’ or amateur landlords, who have inherited properties or invested in a small portfolio as an additional income stream. Echoing previous research (DLUHC 2022), interviewees described many instances of *‘landlords who have no idea’* (P10) of their responsibilities with a lack of formal training or accreditation leading to negligent practices including failure to conduct thorough vetting of prospective tenants, failure to carry out property visits and, more broadly, a lack of knowledge on the responsibilities of landlords and what might regarded as diligent landlord practice.

Interviewees recalled engaging with many ‘decent professional people’ living considerable distances from their rented properties who evidently had little idea that their properties had been used for the purposes of cannabis cultivation.

It was no coincidence, as you do checks on the address, the owners live outside of [city]. So, we’ve had doctors, nurses, good, decent, professional people you ring them up saying, ‘do you know about this cannabis farm? Well, do you know about the one that was found there 12 months ago?’ ‘No, I don’t know anything about it’. (P6)

For such landlords, absenteeism was at times coupled with negligent or naïve practices. One interviewee (P6) recounted a case in which a landlord relied on letting agents sending photographs of the property to show all was well. The interviewee recalled having to point out to the landlord that the weather in the photographs did not match the time of year they were sent, causing considerable surprise and embarrassment to the landlord. Such lack of capable guardianship (Cohen and Felson 1979) is exploited by bad actors, including letting agents working complicitly with cannabis cultivators.

Because a lot of landlords don’t live in [city] either, so they wouldn’t necessarily attend the properties, they’d just pass it on to an estate agent or a management company. They’d deal with everything. If someone’s paying their rent on time and there’s no complaints, then they have no reason to go around, basically. (P33)

Essentially, it’s this letting agent that we’ve already done a warrant with, they’re letting it and other properties for a couple who live out of the country. So they’ve emigrated somewhere else and have a group of properties that are just being looked after by this letting agent, and the letting agent is more or less just taking advantage of the fact that they’re out of the country and they’re never going to drive past it. (P19)

### Poor landlord practice

Importantly, interviewees also described instances of landlords whose poor practices were not initially criminal in and of themselves, but which appeared to offer an opportunity for criminals to exploit. One interviewee recounted the following case:

When I interviewed [the landlord] and I was very much of the opinion that whilst he was somewhat complicit, there was more evidence that he was suffering the property to be utilised for the production of a controlled drug because he was a crap landlord. He talked about how he would pull up on a street one day and people would just come out of the properties with cash in hand as their rent and sometimes he wouldn’t know who these people were and he’d have to ask them what property they were paying rent to him for, and he was giving accounts of poor vetting. I think he was giving accounts of no, little to no, tenancy visits, poor tenancy agreements, and he was just effectively a rogue landlord. (P6)

In this instance, a landlord’s ‘crap’ and seemingly chaotic practices – poor vetting, cash-based transactions, non-existent record keeping – created a gateway for criminality and this landlord appears to have both profited from the illegal use of his properties while seemingly being exploited due to his poor landlord practices. In this sense, the landlord is both victim of exploitation and complicit in the illegal act since he is aware of it, profits from it and chooses not to notify the authorities. Here begins the complex spectrum of complicity for landlords in the PRS with some evidently aware that their properties are being used for illegal activities but also arguably having their negligent or poor practices exploited by criminal actors. According to interviewees, such landlords could quickly find themselves unable to *‘get out’* (P36) once they became involved in organised criminal activities.

### Landlord complicity

While some landlords may, as described above, be victims of having their negligence and poor practices exploited by others, interviewees also recounted many instances of landlords being complicit in cannabis cultivation taking place in their properties. This spectrum of complicity included landlords *‘turning a blind eye’* (P18) to cannabis cultivation, to outright involvement with organised crime actors and at times landlords taking lead roles in criminal enterprises. For those landlords understood to be turning a blind eye and playing something of a passive role in criminal activity, the financial pressures of operating a rental property and the lack of enforcement of PRS-related regulations were identified as key factors which explained their complicity.

This is from somebody that’s in the business that told me this. Blind eye, it’s just too easy. There might be money problems after COVID… It’s a lot of money though, £1,700 per calendar month. £5,000 sweetener to start you going… They’d pay rent as usual, and it’s always on time. Landlords don’t always do the checks they might say they do. (P19)

If you’re going to give somebody who’s a private [landlord] a few hundred quid a month, no questions asked, and they’re going to get it cash in hand and they’re going to get it every month, then there’s a lack of visits going on. (P4)

One interviewee recalled a case involving a landlord whose financial pressures motivated him to become involved in cannabis cultivation:

I mentioned the cost of living. So, some of these people have got buy to let mortgages, mortgage rates are going up, they’re reluctant to put rents up because the people that are renting are on a certain income, who then can’t afford the increase. So, [landlords] then become an easy target. In the past I dealt with a bloke, married, both were professionals, bought a massive house in [area], she got pregnant, he lost his job for a short period of time and was approached by someone in the pubs by saying ‘I’m struggling to pay my mortgage’ so he then used the two top bedrooms [of his rental property], put grows in and it was as simple as that. (LA7)

Here again the spectrum of complicity is complex; this landlord has actively partaken in cannabis cultivation but was also an ‘easy target’ for criminal actors seeking to approach him by virtue of his difficult financial and other circumstances. For other landlords, involvement in cannabis cultivation is not an act of desperation or the result of exploitation; it is instead a much more deliberate, rational and pro-active decision, with interviewees arguing that landlords may deliberately develop a portfolio of properties with the specific intention of facilitating illegal activity such as cannabis cultivation. In such instances, landlords may work closely with organised crime groups, collaborate with complicit letting agencies or take a leading role in an illegal enterprise.

We have two bad landlords that have a good-sized [number] of properties at the lower sort of value property wise, but they would have in excess of, I’d say about 120 properties, which is quite a lot. And they are actively engaged with organised criminals… and are motivated enough to facilitate [cannabis cultivation]. (P2)

If you’ve got properties that are undesirable, which is the ones that probably the private [landlords] are renting out to cannabis farms, then it’s a good market for you because you could buy a row of houses on a street, £15,000 each or whatever… and then you rent them out to people who want to use them for non-legitimate means, then you can make a good bit of money. (P4)

Indeed, in some cases, the use of their property represents just one element of a criminal landlords’ involvement in cannabis cultivation and associated criminality.

He’s a landlord, he’s got six properties, three have got [cannabis] grows and he’s been found with a kilo and a half of spice as well. He’s also been found by Border Force to bring six [irregular migrants] into the country in his motor home illegally. (P27)

Landlord complicity is evidently a key conduit which can facilitate cannabis cultivation since a complicit landlord will ensure that the mechanisms which are at their disposal to help prevent or uncover illegal activity will not be deployed. Such mechanisms may include extensive vetting of prospective tenants, requests for financial transactions to be conducted formally, carrying out regular visits to the property, or following up on concerns raised by local residents. These actions (or lack thereof) taken by landlords ensure the protection of cannabis cultivation sites and serve to remove a potential layer of scrutiny – diligent and scrupulous landlords - for cannabis cultivation actors.

### Letting agent complicity and culpability

Letting agents often play an important and licit part of the management of private rented properties. Interviewees, however, recounted many instances of letting agents being complicit in cannabis cultivation taking place in the PRS, describing them as *‘professional enablers’* (P4) of organised criminal enterprises (see also Levi 2021). As detailed above, complicit letting agents can facilitate the exploitation of landlords who entrust them with the management of their properties. But letting agents may also be involved in more insidious ways and as with the complicity of landlords discussed above, there appears to be a spectrum of criminal involvement for letting agents which once again obscures the extent to which these actors are passively or actively involved in criminality.

In some cases, interviewees recalled letting agents facilitating tenant criminality by paying limited attention to the paperwork required to set up a tenancy

All [prospective tenants] do is, they have their fake ID, which is provided to them, they say that they’re paying cash, so they’ve got no financial records, and they’ll just have like a paper record of the [tenancy agreement], with like a squiggle on it as the signature, they’ll have some fake details down, they’ll have a photocopy of a fake ID; and that’s [the letting agency’s] file for that house. So they, all they’ve had to do is fake an awful paper file and then that is that house legitimately let out to somebody. (P11)

In this instance, the interviewee was confident the agency under discussion was aware of and complicit in cannabis cultivation. However, the agency itself characterised their own practices as incompetent and unprofessional, exercising plausible deniability to claim that their poor practices had been exploited by bad actors. Interviewees often described the facilitation of cannabis cultivation as a *‘side-line’* (P27) for letting agents, requiring them simply to turn a blind eye to criminal activities and eschew their professional responsibilities.

You’ve got professional letting agents turning a blind eye to massive cannabis growers and making a lot of money out of it… If it’s a letting agency they should be doing the bi-annual checks on a property, they should be doing the due diligence checks and the vetting checks for the individuals on behalf of the landlord. (P4)

Other letting agents however were described as working closely with organised crime groups or even being *‘part of’* (P30) such groups.

We are seeing a letting agency linked to multiple properties, I think it’s fair to say that they are complicit in it and not victimless… They purport to be a legitimate business but are part of that organised crime group or certainly know that they are using fraudulent documents in order to obtain the rental agreements for those properties. (P30)

[Letting agents and organised crime groups] are working together, there’s no doubt about it. (P13)

Such agencies were described as particularly unwilling to support criminal investigation, often by refusing to share data and failing to adhere to legal requests for information until taken to court and ordered to comply. Letting agents were also reported as working alongside other professional enablers, the involvement of whom added further layers of complexity and obfuscation for investigations into potential criminal activity.

The difficulty as well is these letting agents are often working with accountancy firms as well, that can then make sure that their books and things, and finances look a certain way, because the accountancy firms are on board as well. (P13)

Like landlords, letting agents are uniquely positioned in the PRS to exploit their position for criminal purposes. Their legitimate presence in the PRS provides a veneer of professionalism and their expert knowledge of rental markets and ability to circumvent robust crime prevention measures – such as vetting prospective tenants and carrying out regular property visits – represent a key conduit in criminality taking place in the PRS.

### Plausible deniability through obfuscation of identity

Plausible deniability can also be operationalised by the perpetrators obscuring their identity. Such obfuscation may include the creation of false or multiple property management companies to allow landlords ostensibly to remove themselves from the direct management of their properties and, should cannabis cultivation be uncovered, enable the exercise of plausible deniability.

They’re a legitimate letting company and they’re on about their fifth name… They’ll keep changing the name on Companies House and they’ll change a letter in the name… it’s hard because you’re basically chasing shadows. (P14)

There’s always been an issue in terms of limited companies because again it seems to be a pattern that we’re seeing where the sort of landlords who, they’ve got these limited companies, they’ve got hundreds of properties, and it seems to be very much that they can sort of detach themselves from what’s going on but there’s clearly some evidence that they are complicit. (P18)

Obfuscation of offending is also facilitated via the use of ‘cut outs’ by landlords, such as letting agents who landlords may argue are responsible for the day-to-day management of properties and are, therefore, deemed responsible for any criminality taking place in these premises.

[We investigated] these six properties and they all related to a particular landlord… Warrants were done and in the six properties three out the six had grows and gardeners were found in situ. But the gardener [wasn’t] the person on the tenancy agreement and that landlord has got no further action purely because he uses a system where he layers subletting agents. (P16)

### Manufacturing and contesting tenant culpability

One further layer of obfuscation exists in landlord and letting agents creating tenancies using false documents and identities to create difficulties for practitioners seeking to determine exactly who is renting a property. Widespread use of cash-based transactions and the absence of formal agreements or contracts were all seemingly designed to leave little evidential trail: ‘*It’s all cash. Nothing hits the books*,* difficult to trace*’ (P19).

There is a trend within our properties that a lot of movement goes on around properties being moved into other people’s names, donating of money, obviously, around the families, swapping and changing of names, fake IDs; a real like very complex web of changes and moving around and things, which really does make it difficult to unpick. (LA3)

You’ll get landlords who were paid money directly without contracts or agreements. Sometimes they have contracts. Sometimes the contracts are in like names of untraceable people, but they’ll get paid like bulk money upfront. I want to rent your property for three months and I will give you £3,000 to do so. (P3)

Interviewers also gave ample evidence of tenants using the PRS to create cannabis farms. Using a rental property, rented via a fake ID from a poor-quality letting agent or inexperienced landlord, was preferrable to a perpetrator using their own home, where it would be difficult to deny any involvement. Further, using a rental property meant that the perpetrators had no compunction in destroying or damaging a property to accommodate the required farming equipment.

In cases where cannabis farming was detected, respondents indicated that tenant farmers also sought to exercise some degree of plausible deniability by claiming to be victims of exploitation and modern slavery. Interviewees accepted that while they had encountered some instances of exploitation of cannabis growers, they remained sceptical of some claims of exploitation and modern slavery made by tenants/growers apprehended in properties. Interviewees accepted that evidencing modern slavery and exploitation can be challenging, especially in cases of debt bondage in home countries for (illegal) migrants, but they nevertheless argued that many tenants/growers claim to have been trafficked and exploited to evade prosecution. In one instance, an interviewee recalled a tenant claiming to have been exploited and trapped in a property to act as a cannabis grower. However, an analysis of his mobile telephone data showed that he had travelled throughout the city, had attended gyms, restaurants and nightclubs, and appeared to be living an active and hedonistic lifestyle.

These many and varied tactics of obfuscation frequently frustrate practitioners’ efforts to tackle cannabis cultivation in the PRS. Precise identification of offenders is often challenging but even when practitioners are confident of actors’ complicity, evidencing this can prove extremely difficult, allowing landlords and letting agents to exercise plausible deniability by pointing to other actors involved in a criminal enterprise and claiming they have been victimised. This often leaves practitioners in a cycle of arresting and prosecuting so-called low hanging fruit such as cannabis growers arrested in properties.

### Limited resources for enforcement, prosecution and supervision

The PRS is subject to numerous laws and regulations and landlords themselves required to meet wide-ranging obligations but these are under-enforced and the PRS is, broadly speaking, under-policed. This means that there are limited resources to undertake the more complex investigation, and where culpability is difficult to prove to criminal standard. One interviewee had recently been engaged in a major criminal case against a landlord with tens of properties and where cannabis farming had taken place only in some. He frankly admitted:

If I do it again, I’ll get a lot of grief from my bosses. It’s been so time consuming. We had to employ someone extra, just to do the admin and paperwork for it all. It is the biggest job we’ve ever done in our office. And that’s just the context of how hard it is to prove the landlord involvement. It is so difficult. (P18)

According to interviewees, the ongoing cuts to funding and lack of resources for practitioners contributes to siloed working practices in which information is not shared effectively with local and regional partners and expertise and community contacts are dispersed. In the context of cannabis cultivation taking place in the PRS, this siloed working appears to create fractures in joined-up approaches and, again, affords opportunities for criminal actors.

One particular landlord… it was a different team every time there was something happening. So, this guy, we didn’t… join the pieces up. This particular individual was an older chap. Very, very engaging, very convincing. Every time they found drugs in a property, one of his properties, it will be a different team, so you didn’t have the same team doing it. The stock answer, every single time we found later, was, ‘Oh my stars, I’m a victim of crime here. What’s gone off here?’ Then the next time it happened, it might be another team that’s come across it or a proactive unit. It wasn’t the same geographical team managing it, so they’re hearing the same thing again. For quite some time, it’s the same excuse, and you might accept it the first time [you hear it]. (P19)

It is easier to create uncertainty around culpability where statutory agencies are not routinely sharing data. As one interviewee reflected, *‘we lose jobs sometimes because we’re not looking at connecting dots’* (P16).

### The disposable nature of poor-quality rentals

While the PRS acts as a facilitator of criminal activities, it is also worth noting the instrumental role of residential property itself as a conduit for cannabis cultivation. The case study area had many pockets of deprivation, where landlords were achieving low rents, failing to invest in property improvement or even maintenance, and often had voids. Respondents were quick to point out that cannabis farming can happen in any type of property, but poor-quality terraces in deprived neighbourhoods constituted a more profitable asset via cannabis farming than legitimate rental. Neighbourhoods of identical properties brought their own affordances for cannabis farmers operating at scale:

What they’ll do is they’ll identify a type of property, and without stereotyping but probably right to do, it’s either a three- or four-storey terraced house that’s got an identical layout to other ones they’ve done. So, they can use the same electrical boards, they can use the same venting, they can use everything. So, they can just go in and they put the model in straight away. (P5)

In these circumstances, landlord complicity with cannabis farming evidenced their willingness to accommodate extensive property damage, since these were properties that were not accruing any capital gain. Landlord complicity was much less likely in locations where house values were buoyant and increasing, and where property damage caused substantial capital loss.

## Discussion and conclusion

While extant research detailing the rise of domestic cannabis cultivation across the world has offered accounts of how and why this import substitution has occurred (Decorte 2010; Barratt et al. 2012), little attention has been paid to the PRS as an important setting in which such criminal activity takes place. The accounts of law enforcement and other practitioners detailed in this article suggest that the PRS presents a series of affordances to individuals willing to engage, either as passive or active participants, in cannabis cultivation in residential properties.

The PRS contains, in theory, a series of actors and mechanisms, which ought to guard against criminal misuse of residential properties. Landlords, letting agents, police and local authorities all represent individuals and organisations which are well positioned to disrupt criminality taking place in the PRS. However, for various reasons, these actors often fail to deliver capable guardianship (Cohen and Felson 1979) of properties in the PRS, creating an environment in which criminality – and particularly cannabis cultivation – is incubated and facilitated. Landlords and lettings agents are empowered to vet potential tenants, create formal (and traceable) agreements with tenants, and conduct regular property checks, all of which ought to create obstacles for criminal endeavours. Instead, for many landlords and letting agents, a key facilitator of criminal activity is their failure to take any of these actions, ensuring activities such as cannabis cultivation are allowed to prosper and attempts by law enforcement and other actors to intervene are frustrated. For the police and local authorities, ongoing financial cuts, dwindling resources and increased demand for service means the PRS is under-policed and opportunities for effective joined-up working are missed as practitioners appear inclined towards retrenchment and siloed working practices.

The complicity of landlords in particular is an opaque and complex facet of cannabis cultivation in the PRS. This spectrum of complicity may include some landlords being both offenders and victims insofar as they turn a blind eye to suspected illegal activity while also finding themselves in the grip of organised crime groups. Poor landlord practices may not always necessarily be akin to complicity in criminal enterprises, but it does appear that landlords at times open themselves up to exploitation due to their poor practices. Understood in this way, poor landlord (and letting agent) practices may be viewed as a gateway to serious criminality, which sees criminal actors exploiting mediocre landlords and leaving landlords (and letting agents) straddling an offender-victim identity. Letting agents, similarly, appear to operate on a spectrum of complicity, sometimes working with criminal landlords and at other times exploiting landlords’ absenteeism, naivety or other disengagement with the management of their properties. ‘Amateur’ landlords are routinely advised to let via a letting agent, as a strategy for compensating for inexperience in the market, and for ‘peace of mind’ that the letting will be managed responsibly (Kale 2025).

Landlords and letting agents are important parts of a functioning PRS (MHCLG 2024) but their legitimate presence in this space allows criminal actors to hide in plain sight and facilitate criminality without immediately raising suspicion. Similar arguments have been made in the context of white-collar crime, in which white-collar criminals can exploit their otherwise legitimate presence to facilitate their offending (see, for example, Gottschalk 2021). Further, like white-collar criminals, landlords and letting agents are uniquely positioned to exploit the criminal potential of the relationships and space in which they operate. In the context of cannabis cultivation in the PRS, landlords and letting agents own or manage the key commodity in this process: the property itself. They can also draw on their expert knowledge of local rental markets and communities, targeting neighbourhoods where residents are unlikely to report suspicious activity to the police for various reasons, where transient and short-term tenancies are not uncommon and where residents may be uninterested in getting to know their neighbours.

The spectrum of complicity for actors such as landlords and letting agents is closely linked to their capacity to exercise plausible deniability for criminal activities. It may even be argued that plausible deniability represents one of the principal reasons why the PRS presents optimal opportunities for crime. Interviewees in this study described three layers of deniability in the context of cannabis cultivation in the PRS: landlords can blame letting agents and tenants; letting agents can blame tenants; and tenants acting as growers can claim to be victimised by organised crime groups or other criminal actors. These denials and obfuscations present significant challenges for agencies seeking to isolate culpability and take enforcement action.

Critically, many of the affordances described in this article are not present or are greatly reduced in owner-occupier and social housing contexts. Owner-occupiers evidently cannot exercise plausible deniability for criminal activities taking place in their homes while they live there and cannot demonstrate that other people have routine use of the property. In the social housing sector, allocation of housing is much more tightly controlled by local authorities and housing associations than the instances described above involving private sector landlords and letting agents. The use of fake identification and the presence of ‘cut out’ agents such as letting agents is limited. Cash-based transactions and the absence of formal agreements are virtually non-existent, unless social housing tenants themselves illegally sublet their properties, which is known to take place (Spencer et al. 2020). However, in the social housing space, such practices are at greater risk of discovery than in the PRS since local authorities are more likely to conduct regular tenancy checks and property visits than in the PRS. Capable guardianship of properties in the social housing sector is, therefore, much more likely to be present and, although not flawless, mechanisms to uncover criminal activity taking place in social housing premises are more consistent and better resourced as compared to the PRS. Indeed, in a ‘good practice guide’ on tackling drug use in rented housing produced by the Home Office, it was recommended that ‘social landlords should provide services to private landlords, such as practical housing management advice, referencing and advice’ (Home Office, no date: 7).

The type of interface between the ‘legal’ and the ‘illegal’ (see Passas 2003; van Duyne et al. 2015) described in this article, and the role of letting agents, in particular, as key enablers of cannabis cultivation, raise important questions about regulation and accountability within the PRS as well. While licensing and oversight mechanisms have increasingly been applied to landlords in recent years, the regulation of letting agents has evolved in a more piecemeal manner such as being legally required to join a government-approved redress scheme, and the introduction of the Tenant Fees Act in 2019 that further curtailed their ability to impose excessive charges on tenants. The enforcement of these regulatory frameworks is fragmented and inconsistent, divided between different jurisdictions, including the National Trading Standards and local authority housing teams, which have been severely affected by ongoing resource constraints for more than a decade (see, for example, Raine et al. 2015; National Audit Office 2021). In addition, there is limited evaluation of how effectively these regulations are implemented or whether they improve management standards, and it would not be an exaggeration to suggest that letting agents in the UK often remain in a regulatory blind spot. This disparity constitutes a regulatory ‘criminogenic asymmetry’, to use Nikos Passas’s (1999) words; an asymmetry that generates incentives and rationalisations for people (and companies) to engage in illegal practices, enables some letting agents to facilitate or, at best, overlook cannabis cultivation (as well as other illegal activities and markets) without the fear of action from law enforcement or regulatory bodies, and reduces the authorities’ ability to control illegality (Passas 1999). In several cases, as our interview data suggests, agents were either complicit or deliberately negligent, hiding behind claims of incompetence while continuing to profit from rents.

Relatedly, it is also likely that much criminality in the PRS, including cannabis cultivation, takes place in disadvantaged communities which experience many and varied indicators of vulnerability as well as high levels of residential transience (Rugg, in press). The spatiality of the PRS is therefore a key explanation of why certain neighbourhoods and properties are targeted by criminal actors for the purpose of cannabis cultivation. These are locations where legitimate renting models offer limited, if any, profit. Previous research has argued that communities experiencing forms of precarity, deprivation and vulnerability may have insular cultural norms, a ‘mind your own business’ attitude and a reluctance to engage with the police (Campbell 1993). High rates of residential mobility also characterise many of these neighbourhoods, echoing Burgess, Shaw and McKay’s classic sociological works on zones of transition and the absence of social cohesion and organisation in deprived urban areas (Burgess 1928; Shaw and McKay 1942). These are all factors which may support and incubate criminal activity such as cannabis cultivation. It may be argued that in such instances, cannabis cultivation drives a downwards cycle during which properties are physically degraded by cultivation; leading to communities suffering from reputational damage; leading to reduced property values, lower rental yields and increased temptation to resort to criminal landlord practices. This cycle further disadvantages residents and communities and reinforces existing ‘mind your own business’ attitudes. Of course, such processes, while damaging already vulnerable communities, may in fact support more cannabis cultivation activity; as one interviewee reflected, *‘it’s just too easy’.*

To conclude, key to our argument is that the PRS is both a set of relationships and a physical space. It is the manipulation of these relationships that makes criminal use of the space possible, and which indicates that crime and housing tenure offers a fruitful area for further investigation. While some extant research has explored the relationship between crime and tenure (see Farrall et al. 2016; Bottoms and Wiles 1986; Livingstone et al. 2014), a narrower focus on the PRS (and, perhaps, organised crime) may deliver important new insights to develop understandings of criminality in this context.

## Data Availability

No datasets were generated or analysed during the current study.

## Footnotes

1 key housing statistics are collected separately for different parts of the UK.

2 It is not clear if this figure includes estate agencies that also manage property.

3 Where data is presented below, police respondents are referred at as P1/2/3, and local authority respondents as LA1/2/3.
